# Impact of COVID-19 on life expectancy at birth in India: a decomposition analysis

**DOI:** 10.1186/s12889-021-11690-z

**Published:** 2021-10-21

**Authors:** Suryakant Yadav, Pawan Kumar Yadav, Neha Yadav

**Affiliations:** 1 Department of Development Studies, International Institute for Population Sciences (IIPS), Mumbai, 400088 India; 2 Centre of Social Medicine and Community Health, Jawaharlal Nehru University (JNU), New Delhi, 110067 India

**Keywords:** Age pattern, Mortality, Sex difference, Life expectancy, Inequality, Age at death, SARS-CoV-2, COVID-19, Pandemic, Gini

## Abstract

**Background:**

Quantifying excess deaths and their impact on life expectancy at birth (e_0_) provide a more comprehensive understanding of the burden of coronavirus disease of 2019 (COVID-19) on mortality. The study aims to comprehend the repercussions of the burden of COVID-19 disease on the life expectancy at birth and inequality in age at death in India.

**Methods:**

The mortality schedule of COVID-19 disease in the pandemic year 2020 was considered one of the causes of death in the category of other infectious diseases in addition to other 21 causes of death in the non-pandemic year 2019 in the Global Burden of Disease (GBD) data. The measures e_0_ and Gini coefficient at age zero (G_0_) and then sex differences in e_0_ and G_0_ over time were analysed by assessing the age-specific contributions based on the application of decomposition analyses in the entire period of 2010–2020.

**Results:**

The e_0_ for men and women decline from 69.5 and 72.0 years in 2019 to 67.5 and 69.8 years, respectively, in 2020. The e_0_ shows a drop of approximately 2.0 years in 2020 when compared to 2019. The sex differences in e_0_ and G_0_ are negatively skewed towards men. The trends in e_0_ and G_0_ value reveal that its value in 2020 is comparable to that in the early 2010s. The age group of 35–79 years showed a remarkable negative contribution to Δe_0_ and ΔG_0_. By causes of death, the COVID-19 disease has contributed − 1.5 and − 9.5%, respectively, whereas cardiovascular diseases contributed the largest value of was 44.6 and 45.9%, respectively, to sex differences in e_0_ and G_0_ in 2020. The outcomes reveal a significant impact of excess deaths caused by the COVID-19 disease on mortality patterns.

**Conclusions:**

The COVID-19 pandemic has negative repercussions on e_0_ and G_0_ in the pandemic year 2020. It has severely affected the distribution of age at death in India, resulting in widening the sex differences in e_0_ and G_0_. The COVID-19 disease demonstrates its potential to cancel the gains of six to eight years in e_0_ and five years in G_0_ and has slowed the mortality transition in India.

## Introduction

Quantifying excess deaths and their impact on life expectancy at birth (e_0_) provide a more comprehensive understanding of the burden of coronavirus disease of 2019 (COVID-19) on mortality [1–6]. Since its beginning in December 2019 in Wuhan, China [7], the toll of deaths and socio-economic losses worldwide are apparent [8–11]. India is one of the countries experiencing excess mortality caused by COVID-19 and has more than 10.3 million confirmed cases and 1.49 lakh deceased cases of COVID-19 pandemic disease [12] in its first wave in the pandemic year 2020. This toll of deaths is almost one-third of that in the USA and half of that in Brazil. India ranks at the third position globally in terms of the toll of deaths attributable to COVID-19 disease in 2020. The case fatality rate (CFR) of COVID-19 deaths is 1.4% in India versus 2.8% in Brazil and 1.8% in the USA in 2020 [13, 14]. These heterogeneities in deaths are also related to the disparity in e_0_ as well as inequality in age at death (G_0_) [15–17]. Analysing the burden of COVID-19 disease based on mortality patterns are critical for understanding the long-term repercussions of the advances in mortality transition in a country. Andrasfay and Goldman [18] demonstrated that the effect of COVID-19 on mortality is large enough for reversing over ten years of progress in closing the black-white gap in e_0_ in the USA. India is one of the countries with an unparalleled convulsion caused by COVID-19 disease. Unravelling the impact of COVID-19 disease on mortality patterns highlights mortality consequences during the first wave of the COVID-19 pandemic in India.

India has past experiences of the toll of deaths caused by infectious diseases such as the Influenza pandemic in 1918 and the Smallpox epidemic in the latter half of the twentieth century [19]. Influenza pandemic manifested burden of at least twelve million deaths in India [20]. Later, India was one of the major reservoirs of Smallpox cases [20, 21]. The epidemiological transition has been apace by controlling deaths from endemic diseases through vaccination and immunisation programmes, better sanitation and housing, and social welfare programs [22–25]. The eradication of smallpox epidemics in 1977 concluded a significant achievement for infant and child mortality decline [26]. The global and native’s health system practices were improvised and implemented. As a result, infectious diseases were contained, establishing low morbidity and mortality [27] caused by killer infectious and parasitic diseases [28, 29]. The pandemic of COVID-19 disease is the latest experience of high morbidity and high mortality worldwide [30, 31].

The progress in mortality transition in India, as measured by the changes in e_0_ and G_0_, is impressive. Despite the burden of infectious disease in India in the twentieth century and currently, with the intrusion of noncommunicable diseases in the mid-1990s [32], the e_0_ for men and women was respectively 22.6 and 23.3 years, in 1911–1921 [33, 34] and increased respectively to 66.9 and 70.0 years in 2011–2015 [35]. However, notably, Malaker and Roy [34] demonstrate hardly any increase in e_0_ between 1901 and 1911 and 1911–1921, a decade witnessing the Influenza pandemic of 1918. Considering the average increase in the e_0_ was of 2.5 years per decade during the period of five decades between 1901–1911 and 1941–1951, the decade of 1911–1921 witnessing the Influenza pandemic of 1918 did not show an increase in e_0_ when compared from the decade of 1901–1911. The decade of 1911–1921 was deeply affected by the loss of lives as it was not reckoned in e_0_.

The share of COVID-19 deaths in 2020 is nearly 1.6% in India as compared to 12 and 14% in the USA and Brazil, respectively,^1^ of the total deaths in the non-pandemic year 2019 [36]. It implies that the distribution of age at deaths by quinquennial age groups of COVID-19 disease is thin compared to the distribution of age at death of overall mortality in India. Hence, the distribution of COVID-19 deaths is quite different from that of the mortality pattern provided in the Sample Registration System (SRS) or Census of India. Therefore, the method of calibration is not applicable. From a methodological point of view, data constraints are apparent.

The appropriate methods would consider the COVID-19 deaths as one of many causes of death on account of excess mortality in the pandemic year 2020 and aggregates to a total number of deaths [4, 37]. Many studies have analysed life table estimates considering the mortality pattern of COVID-19 disease as one of the causes of death. They show that the pandemic disease has the potential to reduce e_0_ by more than one year in the USA and England and Wales to 2.28 years in Madrid [18, 38–40]. A significant impact of COVID-19 disease on life table estimates also points out that with the loss in e_0_, the differences in population subgroups such as sex differences (males minus females) in e_0_ and G_0_ might have reversed [41]. The sex differentials in mortality highlight a significant contribution of adult-age mortality followed by old-age mortality in the twenty-first century compared to the dominance of infant and childhood mortality during the twentieth century [42–44].

Studies analysing mortality patterns concerning COVID-19 are limited in India. Nevertheless, the study fills the knowledge gap by analysing the mortality pattern of COVID-19 disease as one of the causes of death in 2020. The study examines a change in life table estimates of e_0_ and G_0_ in the entire period of 2010–2020, focusing on the non-pandemic year 2019 versus the pandemic year 2020. It assesses the age-specific contributions to sex differences in e_0_ and G_0_ to understand the role of many age groups by sex and over time. Overall, the study aims to comprehend the repercussions of the burden of COVID-19 disease on the life expectancy at birth and inequality in age at death in India.

## Methods

### Data

We retrieved data between 30 Jan 2020 and 31 Dec 2020 from COVID19-India Application Programming Interface (API) portal available in the public domain [12]. It provides data on a daily basis up to the district level since the first date, 30 Jan 2020, a COVID-19 case was found in India. Data on COVID-19 cases on this portal is updated from state bulletins, official handles, PBI, Press Trust of India (PTI), and Asian News International (ANI) reports. The distributions of death and COVID-19 cases provided by COVID19-India API and Ministry of Health and Family Welfare (MoHFW) are very similar (Table 1) [45, 46].

**Table 1:** Distribution of deaths by broad age groups, MoHFW and COVID19-India API, 2020

Age group	MoHFW, GOI^a^		COVID19-India API^b^	
	Deaths (%)	Cases (%)	Deaths (%)	Cases (%)
**< 10**	0.27	2.97	0.23	2.60
**10–20**	0.53	8.50	0.45	5.01
**20–30**	2.08	19.35	2.60	15.91
**30–40**	5.27	21.15	6.55	19.93
**40–50**	11.98	17.50	15.03	19.92
**50–60**	23.29	15.07	26.29	20.88
**60–70**	28.76	9.99	26.44	11.14
**70–80**	19.99	4.19	16.21	3.83
**> 80**	7.82	1.28	6.22	0.78
**Sample**	83,189	4,938,845	21,277	161,727

Source: ^a^ [45, 46], ^b^Own calculations [12]

**Fig. 1:**
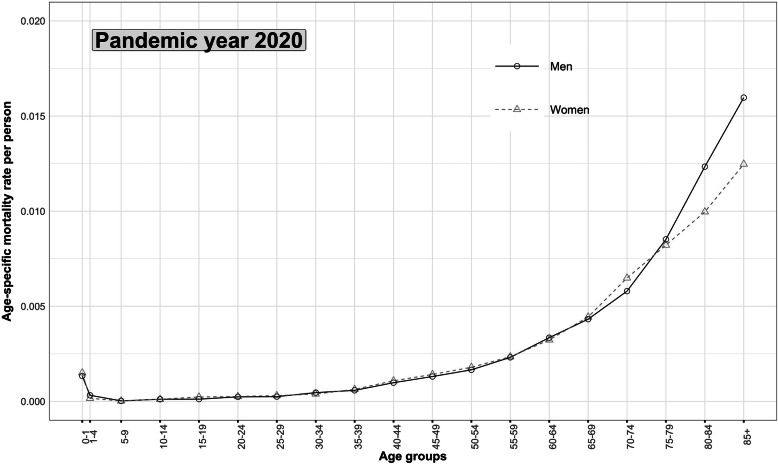
Age pattern of mortality of COVID-19 disease, India, Jan to Dec 2020

There is a plausibility of underreporting and missing cases of COVID-19 disease [47] while collating COVID-19 data by various government and non-government organisations. The missing and unidentified cases or infections warps the mortality estimates of COVID-19 disease [17, 48–53]. Nevertheless, the second and third seroepidemiological surveys in India estimated overall adjusted prevalence of 6.6 [54] and 14.3% [55] infections, respectively, using Abbott IgG assay. The first seroepidemiological survey shows an estimated seroprevalence of 0.73% using ELISA assay [56]. The seroepidemiological survey differs among themselves by use of assays [57], indicating huge asymptomatic carriers in the studied period ([58], Fig. 1). O’Driscoll, Ribeiro Dos Santos [59] demonstrates the consistency of seroprevalence in seroepidemiological surveys and age distribution of deaths in young population across 45 countries, whereas considerable heterogeneity in fatality rates, especially in old ages. We considered the projected population until December 2020 [60] and an average seroprevalence of 7.21% for adjusting COVID-19 infections by broad age groups and sex on a pro-rata basis ([4, 54]: Table 2). Studies have demonstrated no significant differences in seroprevalence across age groups [54, 57, 61, 62]. However, a variation in overall seroprevalence in children, adults, and the generalised population is noted across national, regional, and local studies [63–66]. Given that, the seroprevalence estimate in 0–9 years is assumed at 5.4%, which is the same as that of 10–17 years in India [67]. The total number of infections in the pandemic year 2020 is calculated at 94.21 million. This study used death and confirmed cases and estimated infections of COVID-19 disease in the studied period to calculate adjusted age-specific infection fatality rates (ASIFR).

**Table 2:** Life table estimates, India, 2014–18, 2019, 2020

Age groups	SRS (2014–18)^b^		Non-pandemic year (2019)^a^		Pandemic year (2020)^a^	
	Male	Female	Male	Female	Male	Female
**0–1**	68.2	70.7	69.5	72.1	67.5	69.8
**1–4**	69.8	72.5	70.6	73.4	68.7	71.2
**5–9**	66.1	69.0	66.9	69.8	65.0	67.6
**10–14**	61.3	64.2	62.1	65.0	60.2	62.8
**15–19**	56.5	59.4	57.3	60.2	55.4	58.0
**20–24**	51.7	54.6	52.5	55.5	50.7	53.3
**25–29**	47.0	49.9	47.8	50.8	46.0	48.7
**30–34**	42.4	45.2	43.2	46.1	41.5	44.1
**35–39**	37.8	40.5	38.7	41.4	37.0	39.5
**40–44**	33.4	35.8	34.3	36.8	32.7	34.9
**45–49**	29.1	31.3	29.9	32.2	28.4	30.5
**50–54**	24.9	26.9	25.8	27.8	24.4	26.2
**55–59**	21.0	22.8	21.8	23.6	20.6	22.3
**60–64**	17.4	18.9	18.2	19.7	17.1	18.4
**65–69**	14.1	15.3	14.8	16.0	13.9	15.0
**70–74**	11.1	12.1	11.8	12.7	11.0	11.9
**75–79**	8.5	9.2	9.2	9.9	8.6	9.2
**80–84**	6.2	6.6	7.1	7.6	6.6	7.1
**85+**	4.5	4.7	5.5	5.8	5.1	5.4

a: Own calculations. b: Sample Registration System (2014–2018)

There are chances of misclassification of causes of deaths because of comorbidities and lack of medical care facilities with true records. Woolf, Chapman [37] demonstrated misclassification of causes of death including COVID-19 disease in the USA [3], using Joinpoint regression analysis on weekly mortality data. Particularly in India, the age-specific death rate (ASDR) of many causes of death are not yet reported until new updates are released for the pandemic year 2020 from the Global Burden of Disease (GBD) or Office of the Registrar General & Census Commissioner (ORG&CC). So, in the lack of the latest data, we considered 21 causes of death data available for the previous year 2019 from Global Burden of Disease (GBD) [36].

We assumed the same age pattern of mortality for these 21 causes of death in 2020. Given that, we considered COVID-19 disease as one of the causes of death in the category of other infectious diseases [68] in 2020. So, in total, we considered 22 causes of death and then computed the overall age-specific death rate in 2020. The assumption of the same age pattern of mortality in the pandemic year 2020 may not be strictly correct. However, mortality rates would slightly decline given the previous trends but got disrupted in this pandemic time. It can be only argued that if road accidents reduce in lockdowns [69, 70], then, at the same time, heart strokes may result in more deaths in the lack of medical care facilities [71–73].

### Demographic and statistical techniques

#### Construction of abridged life tables

We constructed abridged life tables for the entire period of 2010–2020 based on the mortality rates of many causes of death by sex. The SRS (2014–18) based abridged life tables are also used for comparison [35]. Chiang [74] method is based on the derivation of relation for the total number of person-years lived between exact ages *x* and *x + n* (_*n*_L_x_) in terms of the average number of years lived by an individual of age *x* who dies in the interval (*x, x + n*) (_*n*_a_x_). The columns of the life table are obtained using the following formulas:

_*n*_M_x_: observed mortality rate,

_*n*_q_x_: probability of dying between age *x* and *x + n*

$$ {{}_n\mathrm{q}}_{\mathrm{x}}=\frac{n\ast \left({{}_n\mathrm{M}}_{\mathrm{x}}\right)}{1+\left(n-{{}_n\mathrm{a}}_{\mathrm{x}}\right)\ast {{}_n\mathrm{M}}_{\mathrm{x}}}, $$

l_x_: number of people alive at the exact age *x* among a hypothetical birth cohort of 100,000, usually the radix of the life table

$$ {\mathrm{l}}_{\mathrm{x}+\mathrm{n}}={\mathrm{l}}_{\mathrm{x}}\ast \left(1-{{}_n\mathrm{q}}_{\mathrm{x}}\right), $$

_*n*_L_x_***:*** total number of person-years lived between exact ages *x* and *x + n*

$$ {{}_n\mathrm{L}}_{\mathrm{x}}=n\ast \left({1}_{\mathrm{x}}-{{}_n\mathrm{d}}_{\mathrm{x}}+{{}_n\mathrm{a}}_{\mathrm{x}}\ast {{}_n\mathrm{d}}_{\mathrm{x}}\ \right), $$

_*n*_d_x_***:*** number of deaths in the age interval *x* to *x + n*

$$ {{}_n\mathrm{d}}_{\mathrm{x}}={1}_{\mathrm{x}}\ast {{}_n\mathrm{q}}_{\mathrm{x}}, $$

T_x_: total number of person-years lived beyond age *x*

$$ {\mathrm{T}}_{\mathrm{x}}={\mathrm{T}}_{\mathrm{x}+\mathrm{n}}+{{}_n\mathrm{L}}_{\mathrm{x}}, $$

e_x_: average number of years of life remaining for a person alive at the beginning of age interval x

$$ {\mathrm{e}}_{\mathrm{x}}=\frac{{\mathrm{T}}_{\mathrm{x}}}{1_{\mathrm{x}}}. $$

### Measuring inequality in life expectancy

#### Gini coefficient at age zero/at birth (G_0_)

The Gini coefficient (G) measures inequality in age at death or disparity in life span. It is a better measure for understanding the age-specific contributions than that of e_0_ [42]. The Gini coefficient reflects the changes in adult mortality sufficiently and is not extremely sensitive to infant and child mortality decline [75]. The Gini coefficient value ranges between 0 and 1. It is equal to 0 if all people die at the same age and is equal to 1 if an individual dies at age zero, and one individual dies at an infinitely old age. The higher or lower value of the Gini coefficient shows a higher or lower magnitude of inter-individual differences in length of life [75].

According to Hanada (1983) [76], the Gini coefficient at age *x* (G_x_) is calculated by the formula

$$ {G}_x=1-\frac{1}{e_0{\left[{l}_0\right]}^2}\underset{0}{\overset{\infty }{\int }}{\left[{l}_x\right]}^2. $$

The following above equation [42, 75] is used for the calculation of the Gini coefficient at birth/age zero (G_0_) from the abridged life table

$$ {G}_0=1-\frac{1}{e_0{\left[{l}_0\right]}^2}\ast {\sum}_{t=0}^{w-1}\left[{\left({l}_{t+1}\right)}^2+{A}_x^{\prime}\left({\left({l}_t\right)}^2-{\left({l}_{t+1}\right)}^2\right)\right] $$

where,

$$ {A}_0^{\prime }={A}_0\ast \left[1-{q}_0\frac{3+0.831\ {A}_0}{2+{q}_0}\right] $$

$$ {A}_x^{\prime }=\frac{\left[1-\left(\frac{2}{3}\right){q}_x+{C}_x\left(2-{q}_x-\left(\frac{6}{5}\right){C}_x\right)\;\right]\ }{2-{q}_x},\forall x\ge 1 $$

where, $$ {C}_x={A}_x-\frac{1}{2} $$,

$$ {A}_x=\frac{\left(\frac{{{}_nL}_x}{n}\right)-{l}_{x+n}}{l_x-{l}_{x+n}}. $$

### Decomposition of e_0_ and G_0_

#### Decomposition of e_0_ using the discrete method

Arriaga’s [77] discrete decomposition method was used for the decomposition of e_0_. Consider the age group *x* to *x + n* of life Tables 1 and 2, where script ‘1’ refers to the base life table population. The total effect (_*n*_Δ_x_) of a difference in mortality rates between age group *x* to *x + n* on e_0_ between two life tables can be calculated by using Arriaga’s method as

$$ {{}_n\Delta}_{\mathrm{x}}=\frac{l_x^1\ }{l_0}\left(\frac{{{}_nL}_x^2}{l_x^2}-\frac{{{}_nL}_x^1}{l_x^1}\right)+\frac{T_{x+n}^2}{l_0}\left(\ \frac{l_x^1}{l_x^2}-\frac{l_{x+n}^1}{l_{x+n}^2}\right) $$

where, ${l}_x^1$ = number of persons alive at exact age *x* in the life table ‘1’, ${l}_x^2$ = number of persons alive at exact age *x* in the life table ‘2’, ${{}_nL}_x^1$ = number of person-years lived between ages *x* and *x + n* in the life Table 1, ${{}_nL}_x^2$ = number of person-years lived between ages *x* and *x + n* in the life Table 2, ${T}_x^1$ = number of person-year lived above exact age *x* in the life Table 1 (base life table), ${T}_x^2$ = number of person-year lived above exact age *x* in the life Table 2.

The first part of the right-hand side (RHS) of the above formula

$$ \frac{l_x^1\ }{l_0}\left(\frac{{{}_nL}_x^2}{l_x^2}-\frac{{{}_nL}_x^1}{l_x^1}\right) $$

corresponds to the direct effect of a change in mortality rates between ages *x* and *x + n*, i.e. the effect that a change of the number of years lived between *x* to *x + n* produces on e_0_.

The second term of the above formula (8)

$$ \frac{T_{x+n}^2}{l_0}\left(\ \frac{l_x^1}{l_x^2}-\frac{l_{x+n}^1}{l_{x+n}^2}\right) $$

corresponds to the sum of the indirect and interaction effects, i.e. the contribution resulting from the person-years to be added because additional survivors at age *x + n* are exposed to new mortality conditions [78]. We can say that the total contributions of an age group to the life expectancy gap (in years) is the sum of two mathematical terms, first corresponds to the direct effect and seconds to indirect and interaction effects.

#### Decomposition of e_0_ and G_0_ using the replacement method

The formula for the decomposition of differences between the Gini coefficient at age *x* by age was promulgated by researchers [75]. The general procedure for decomposition by age group of a difference in two Gini coefficients *G*_0_ and ${G}_0^{\prime }$ is given as

$$ {G}_0-{G}_0^{\prime }={\sum}_{i=0}^{n-1}\left({\in}_{0,{x}_{i+1}}-{\in}_{0,{x}_i}\right)={\sum}_{i=0}^n{\in}_i $$

A general procedure for the computation of age-specific components of the difference is

$$ {\in}_i={G}_0\left[{M}^{\left({x}_i\right)}\right]-{G}_0^{\prime}\left[{M}^{\left({x}_i\right)}\right] $$

where, ${M}^{\left({x}_i\right)}$ is a vector of age-specific mortality rates with elements *m*′_*x*_ for *x* < = *x*_*i*_ and *m*_*x*_ for *x* > = *x*_*i*_. It determines a stepwise replacement of one mortality pattern by another, beginning from the youngest to the oldest. For any decomposition methods, we have used 2019 as the base year.

## Results

### Age pattern of mortality of COVID-19 disease, the pandemic year 2020

The shape of the age pattern of mortality of COVID-19 disease is similar to a usual age pattern of mortality (Fig. 1). In the infant, child, adolescent, and adult age groups, the ASIFR in men and women is very close. The sex differentials in mortality of COVID-19 disease is apparent in old age groups only. Nevertheless, the slope of the age pattern of mortality, as measured by a Gompertz-Makeham (GM) model [79], is steeper in men than in women [43, 80]. The steep slope of mortality in old ages confirms rapid acceleration in mortality rates in men than in women. Hence, as summarised by the features of the age pattern of mortality, the mortality risk of COVID-19 disease appears higher in men than in women.

### Mortality disruptions in adult and old ages in the pandemic year 2020

**Fig. 2:**
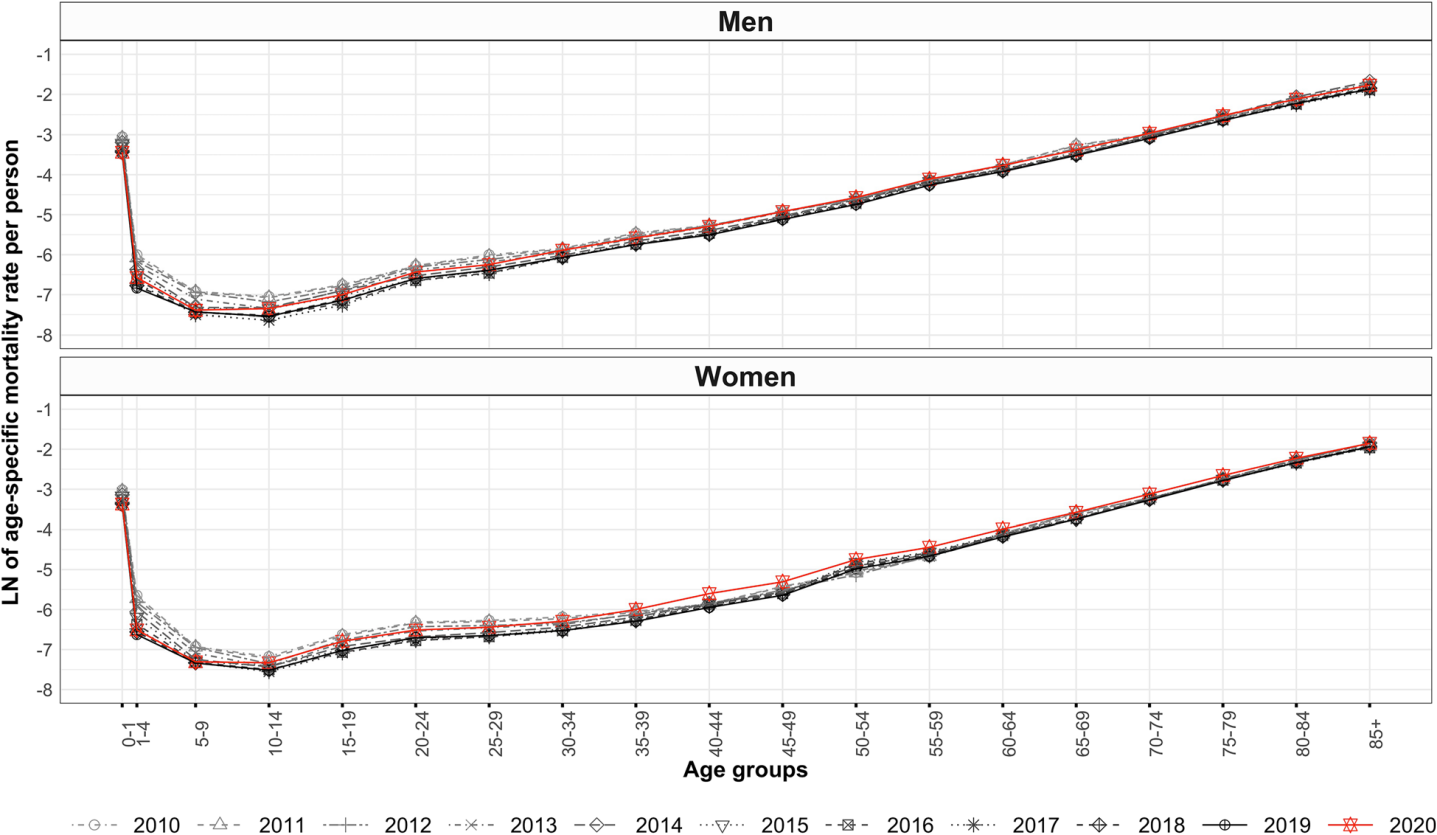
Age pattern of mortality, India, 2010–2020

Figure 2 shows the age pattern of mortality of men and women for the entire period of 2010–2020. A comparison of the age pattern of mortality in these years shows discernible disruptions in adult^2^ and old ages whereas small, subtle changes in infant, child and adolescent ages in 2020.

**Fig. 3:**
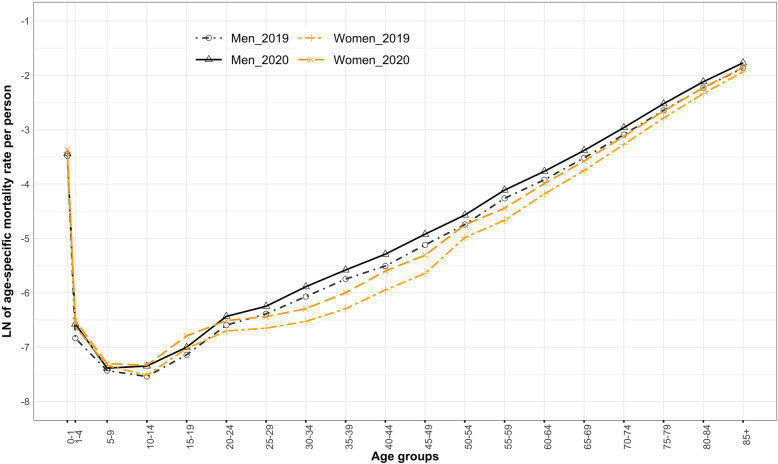
Age pattern of mortality, India, the non-pandemic year 2019 and the pandemic year 2020

Figure 3 shows the age pattern of mortality of men and women in 2019 and 2020. Comparing the age pattern of mortality between two sexes reveals higher mortality rates in many age groups in men than in women. The higher mortality rates in the age groups of adults (20–64 years) and old (65–79 years) in men than in women signify a significant gender gap in mortality between 2019 and 2020. The oldest of olds (80+ years) experiencing the highest mortality rates shows a narrow gender gap in mortality rates.

An increase in adult- and old-age mortality rates is apparent from the slope of the natural logarithm of the exponential curve of mortality rates. There was a noticeable rise in death rates at adult and old ages in 2020 for both men and women. A high adult- and old-age mortality rates is apparent, indicating a wider sex differential in 2020 than in 2019.

### Comparison of life table estimates, the pandemic year 2020 versus previous years

Table 2 shows the life table estimates based on the age pattern of mortality in 2020 and its comparison with previous years 2019 and 2014–18 [35]. Table 2 shows e_0_s for men and women were respectively 69.5 [67.4–71.3]^3^ years and 72.1 [69.9–73.9] years in 2019. However, in 2020, e_0_s for men and women were 67.5 [65.4–69.4] years and 69.8 [67.6–71.7] years, respectively. Comparing mortality estimates between 2019 and 2020 reveals a large difference between the two e_0_s. It is evident that including COVID-19 disease as one of the causes of death led to a drop of 2.0 and 2.3 years in e_0_ for men and women, respectively. The drop in e_65_ attributable to COVID-19 disease is of one year for both men and women. Compared to the sex difference in e_0_ in 2020, the sex difference in e_0_ in 2014–2018 from SRS showed a slightly wider sex differential.

**Fig. 4:**
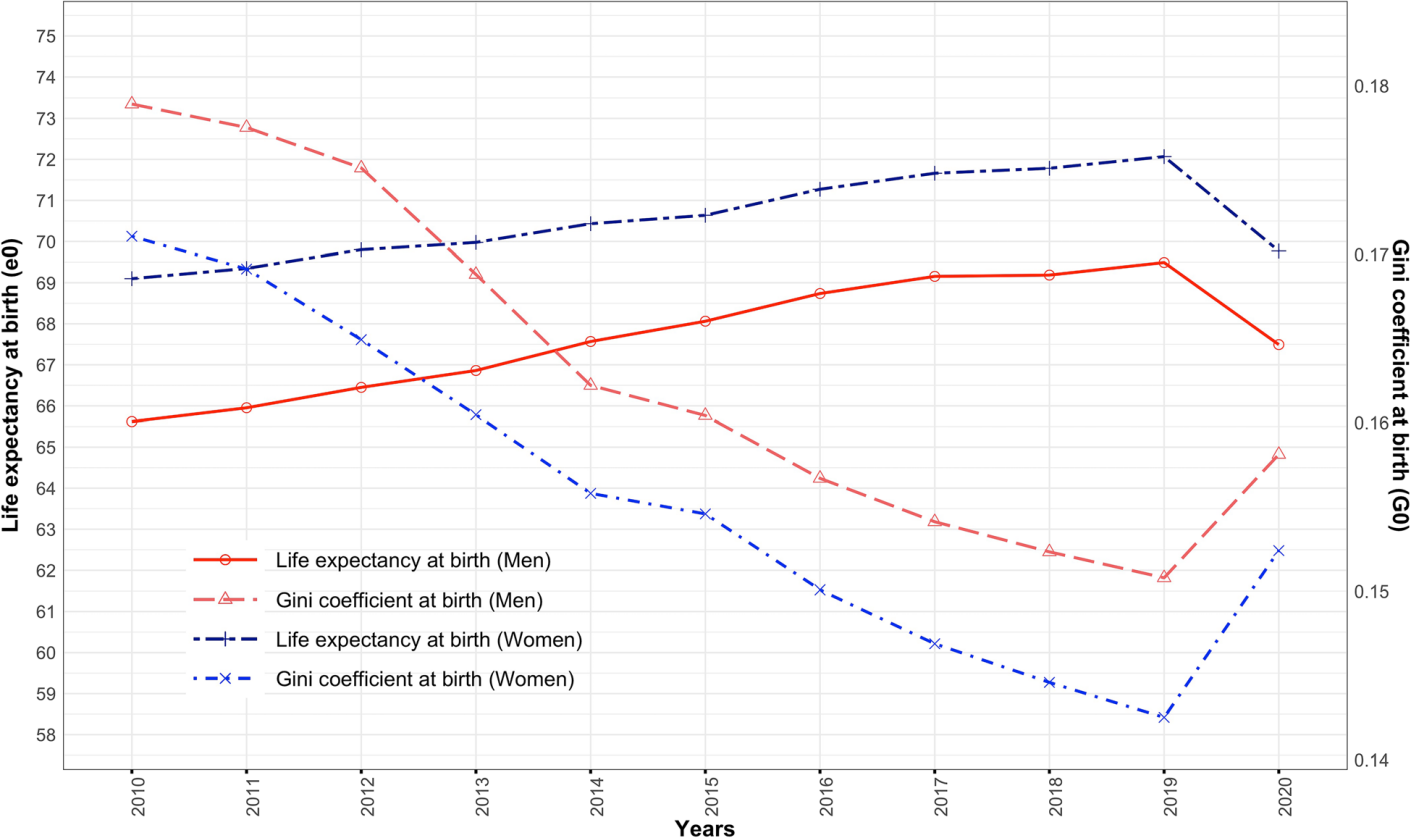
Trends in e_0_ and G_0_, India, 2010–2020

Figure 4 shows the trends in e_0_ and G_0_ for men and women in 2010–2020. The trends in e_0_ reveal that its value in 2020 is comparable to that in the early 2010s. The increase in e_0_ achieved in the past six to eight years is repudiated against a drop of ~ two years. The burden of COVID-19 disease demonstrates the loss of person-years lived and provides a piece of evidence to the reversal of the progress in mortality transition by nearly a decade. The COVID-19 disease shows a potential to cancel a significant gain in e_0_ in 2010–2020.

The trends in G_0_ confirm a rise in disparity or more dispersion in age at death in the pandemic year 2020 compared to previous years in the period of 2010–2020. Thus, a large dispersion in age at death confirms a disproportionate rise in the number of deaths in many age groups attributable to COVID-19 disease. The G_0_ values of men and women were 0.159 and 0.153 in 2020, contributed by a higher mortality rate in adult and old age groups. The trends in G_0_ reveal that its value in 2020 is almost close to its value in 2015. It rolled back to a higher value cancelling its gain in the last five years when compared in the past. The reversal of trends in G_0_ values confirms the excess mortality of COVID-19 disease for an unequal distribution of age at death. In addition to confirming the loss of person-years lived, the results show a significant impact of COVID-19 disease on the inequality in age at death.

### Age-specific contributions to e_0_ and G_0_, India, 2010–2020

Table 3 shows the application of decomposition methods for analysing the age-specific contributions to Δe_0_ by discrete [77] and the replacement [75] methods [82, 83]. The results from both methods are similar. We prefer to show the results based on the replacement methods.

**Table 3:** Age-specific percent contributions to Δe_0_, men and women, India, 2019–2020, replacement and discrete methods

Age Group	Men		Women	
	Replacement method	Discrete method	Replacement method	Discrete method
**0–1**	4.6	4.3	4.6	4.3
**1–4**	4.3	4.1	2.0	1.9
**5–9**	0.4	0.4	0.3	0.3
**10–14**	1.6	1.5	1.5	1.4
**15–19**	1.5	1.5	2.7	2.6
**20–24**	2.8	2.7	2.8	2.7
**25–29**	2.7	2.5	3.0	2.8
**30–34**	4.4	4.1	3.4	3.2
**35–39**	4.8	4.5	5.0	4.6
**40–44**	7.0	6.6	7.3	6.8
**45–49**	7.9	7.5	8.2	7.7
**50–54**	8.2	7.8	8.6	8.1
**55–59**	9.0	8.6	9.0	8.6
**60–64**	9.8	9.4	9.6	9.1
**65–69**	8.9	8.6	9.5	9.2
**70–74**	8.7	8.2	9.0	8.6
**75–79**	6.8	7.0	7.0	7.3
**80–84**	4.3	5.0	4.5	5.1
**85+**	2.9	5.6	2.8	6.0

Source: Own calculations

Tables 4 and 5 show the age-specific per cent contributions^2^ to Δe_0_ for men and women, respectively, from 2010 through 2020. Results show a consistent pattern of the highest contribution of the infant age group to Δe_0_ in the period 2010–2019. However, the infant (0–1 year) age group in males and females only contributed − 4.5 and − 4.6%, respectively, in 2020 compared to larger contributions of infants in previous years. A distinct deviation in the infants and children age group’s contribution against null confirms an effect of COVID-19 disease in the age group of 0–4 years in both sexes. The burden of COVID-19 disease was marginal in the age groups of 5–9 and 10–19 years. Nonetheless, the burden of COVID-19 disease sloped from young-adult (20–34 years) age group in men and women with a contribution of − 9.8 and − 9.1%, respectively, in 2020. The middle-aged adult (35–49 years) age group in men and women showed a higher contribution of − 19.6 and − 20.3%, respectively, maintaining the slope in mortality rates. Men and women show the largest burden of COVID-19 disease in their older adult (50–64 years) age group with a contribution of − 26.4 and − 27.0%, respectively, to Δe_0_. The young-old (65–79 years) age group in men and women showed almost a similar contribution of − 24.2 and − 25.2% to Δe_0_. The oldest of olds (80+ years) age group in men and women showed a small contribution of − 6.3 and − 6.5%, respectively, to Δe_0_. Overall, a significant contribution from the age group of 35–79 years to Δe_0_ confirms the substantial burden of COVID-19 disease, attributable to higher adult- and old-age mortality rates in 2020 than in previous years.

**Table 4:** Age-specific contributions to Δe_0_, men, India, 2010–2020

Year	2010	2011	2012	2013	2014	2015	2016	2017	2018	2020
**0–1**	28.0	28.2	29.8	31.6	35.8	40.3	43.9	56.1	34.7	−4.5
**1–4**	9.4	8.9	8.8	8.4	8.8	8.8	9.2	11.2	6.0	−4.3
**5–9**	3.1	3.3	3.7	2.6	1.1	1.1	−0.2	−3.3	− 0.4	− 0.4
**10–14**	2.4	2.5	2.1	1.2	1.9	2.2	− 0.1	− 4.0	1.1	−1.6
**15–19**	2.5	2.5	2.3	2.2	3.0	1.8	−1.7	−7.0	1.4	−1.5
**20–24**	3.1	3.3	3.5	2.8	1.2	−0.3	− 1.9	− 4.9	− 0.5	−2.8
**25–29**	4.2	4.1	3.5	3.0	1.6	−0.3	− 3.3	−8.9	− 1.4	− 2.7
**30–34**	2.8	2.8	2.8	2.2	1.2	0.4	0.3	−0.2	0.3	−4.3
**35–39**	4.5	4.5	4.0	3.4	2.6	1.7	1.2	0.2	1.5	−4.8
**40–44**	3.9	4.2	4.9	4.9	3.6	0.9	3.3	9.5	4.2	−7.0
**45–49**	4.0	4.3	5.3	5.5	3.4	3.4	6.3	10.3	4.1	−7.8
**50–54**	3.1	3.3	4.1	5.0	5.7	6.8	6.6	5.5	6.8	−8.1
**55–59**	3.2	3.8	4.7	3.3	2.2	7.4	16.0	27.1	8.4	−8.9
**60–64**	5.4	5.9	5.7	2.5	1.6	5.7	8.8	9.9	4.8	−9.7
**65–69**	9.1	9.3	7.6	4.0	1.2	2.5	5.4	7.1	2.6	−8.8
**70–74**	4.4	4.4	4.3	5.0	5.3	4.9	6.4	9.0	7.9	−8.6
**75–79**	3.3	2.8	2.4	5.0	7.1	4.9	2.5	−0.3	7.5	−6.8
**80–84**	2.1	1.3	0.8	3.9	6.6	3.9	−0.3	−5.9	5.7	−4.3
**85+**	1.4	0.4	− 0.3	3.4	6.4	3.6	−2.5	−11.3	5.1	−2.9

Source: Own calculations; base year: 2019

**Table 5:** Age-specific contributions to Δe_0_, women, India, 2010–2020

Year	2010	2011	2012	2013	2014	2015	2016	2017	2018	2020
**0–1**	37.4	37.7	41.4	41.6	44.4	43.2	45.11	51.0	41.8	−4.6
**1–4**	20.3	19.4	19.9	17.8	17.1	14.0	13.54	13.8	9.0	−2.0
**5–9**	3.6	3.7	4.3	2.8	1.2	0.6	0.60	0.5	0.3	−0.3
**10–14**	2.2	2.0	1.3	0.7	0.9	1.2	0.32	−1.4	0.5	−1.4
**15–19**	3.9	3.8	3.1	2.5	1.6	0.4	− 1.33	−3.6	0.0	−2.7
**20–24**	4.8	4.9	4.2	2.6	0.3	−1.1	−2.77	−5.3	− 1.7	− 2.8
**25–29**	4.5	4.4	3.7	3.1	1.4	0.1	−0.87	−2.3	−0.2	−3.0
**30–34**	4.0	3.7	2.7	2.3	1.6	0.8	0.21	−0.8	0.6	− 3.4
**35–39**	3.1	3.0	2.7	3.2	2.3	1.5	1.43	1.2	1.3	−4.9
**40–44**	1.2	1.2	1.0	2.1	2.8	3.0	4.30	6.6	4.2	−7.3
**45–49**	3.9	4.0	3.0	2.6	2.4	2.5	3.28	4.5	3.2	−8.1
**50–54**	− 2.2	−2.7	−4.6	−1.9	3.6	9.1	13.74	19.2	11.2	−8.5
**55–59**	−0.7	−0.2	− 0.1	− 0.1	2.4	6.6	9.47	12.2	7.8	−9.0
**60–64**	0.8	2.1	4.1	2.8	1.9	5.9	8.08	8.4	5.3	−9.5
**65–69**	6.7	8.0	9.3	5.7	0.5	2.3	3.97	3.3	0.7	−9.4
**70–74**	1.7	2.5	3.2	2.4	2.3	4.1	5.03	5.5	5.3	−8.9
**75–79**	1.7	1.6	1.6	3.1	3.9	3.0	1.37	−0.2	4.6	−7.0
**80–84**	1.5	0.7	0.2	3.1	4.3	1.7	−1.36	−4.1	3.3	−4.5
**85+**	1.5	−0.1	−1.0	3.4	5.1	1.0	−4.1	−8.5	2.8	−2.8

Source: Own calculations; base year: 2019

The age-specific contributions to ΔG_0_ for men (Table 6) and women (Table 7) elucidate the disequalising effect and equalising effect of many age groups during 2010–2020. A remarkable disequalising effect of − 41.4 and − 38.8%, respectively, was contributed by men and women in their middle-aged adult age group. Besides, older-adult and young-adult age groups contributed respectively − 27.9 and − 26.8% in men and − 30.9 and − 21.5% in women. In sum, the adult (20–64 years) age group contributed − 96.1 and − 91.2% in men and in women that are distinguishable and larger from age-specific contributions in previous years. This distinguishable contribution of the adult age group confirms a heavy toll of young lives caused by COVID-19 disease disrupting the distribution of age at death in 2020 compared to previous years. Young-old and oldest of old age groups showed equalising effects on ΔG_0_ with a contribution of 17.7 and 18.5% in men and 6.7 and 14.1%, respectively, in women. In the old (65+ years) age group, men compared to women show a larger contribution to ΔG_0_. The contribution of infants, children, and adolescents’ age groups to ΔG_0_ was smaller than that of adult age groups, however, comparable to that of old age groups. Compared to age-specific contributions to Δe_0_, the contribution of infant and child age groups to ΔG_0_ were larger in men than in women.

**Table 6:** Age-specific contributions to ΔG_0_, men, India, 2010–2020

Year	2010	2011	2012	2013	2014	2015	2016	2017	2018	2020
**0–1**	47.4	45.8	45.7	56.4	73.1	72.7	68.32	69.4	83.0	−15.0
**1–4**	15.8	14.4	13.3	14.9	17.7	15.8	14.23	13.8	14.2	−14.1
**5–9**	5.1	5.2	5.5	4.5	2.2	1.9	−0.31	−4.0	−0.9	−1.4
**10–14**	3.9	3.8	3.0	2.0	3.6	3.7	−0.09	−4.6	2.5	−5.0
**15–19**	3.8	3.7	3.2	3.5	5.5	3.0	−2.45	−7.9	3.1	−4.6
**20–24**	4.5	4.6	4.6	4.4	2.1	−0.5	− 2.65	−5.4	−1.0	−8.2
**25–29**	5.9	5.6	4.5	4.5	2.7	−0.4	−4.29	−9.2	−2.8	−7.3
**30–34**	3.7	3.5	3.3	3.1	2.0	0.6	0.36	−0.2	0.6	−11.3
**35–39**	5.5	5.2	4.4	4.5	3.8	2.3	1.42	0.2	2.7	−11.5
**40–44**	4.3	4.4	4.9	5.7	4.9	1.1	3.40	7.8	6.7	−15.1
**45–49**	3.8	4.0	4.5	5.6	4.0	3.6	5.64	7.4	5.8	−14.7
**50–54**	2.4	2.5	2.9	4.2	5.6	5.9	4.90	3.2	7.8	−12.4
**55–59**	1.8	2.0	2.4	2.0	1.6	4.7	8.68	11.8	7.2	−9.8
**60–64**	1.5	1.6	1.5	0.9	0.7	2.0	2.70	2.5	2.4	−5.7
**65–69**	−0.4	−0.4	−0.2	0.0	0.0	0.1	0.10	0.1	0.1	0.4
**70–74**	−1.8	−1.7	−1.6	−2.0	−2.2	−1.8	−2.08	−2.3	−3.7	6.7
**75–79**	−2.7	−2.2	−1.7	−4.1	−6.5	−4.0	−1.74	0.2	−7.9	10.6
**80–84**	−2.4	−1.5	−0.8	−4.7	− 9.2	−4.8	0.32	4.8	−9.1	9.8
**85+**	−2.1	−0.6	0.5	−5.4	−11.6	− 5.8	3.5	12.5	−10.7	8.7

Source: Own calculations; base year: 2019

**Table 7:** Age-specific contributions to ΔG_0_, women, India, 2010–2020

Year	2010	2011	2012	2013	2014	2015	2016	2017	2018	2020
**0–1**	46.3	45.9	49.8	57.5	64.7	60.8	56.34	56.3	67.2	−12.7
**1–4**	24.9	23.4	23.7	24.4	24.7	19.5	16.76	15.1	14.4	−5.4
**5–9**	4.3	4.4	5.0	3.8	1.7	0.8	0.72	0.5	0.5	−0.8
**10–14**	2.6	2.3	1.4	0.9	1.2	1.6	0.38	−1.4	0.7	−3.8
**15–19**	4.5	4.3	3.5	3.2	2.2	0.5	−1.54	−3.6	0.0	−6.9
**20–24**	5.3	5.4	4.6	3.2	0.4	−1.3	−3.10	−5.3	−2.4	− 6.9
**25–29**	4.8	4.7	3.8	3.7	1.7	0.1	−0.94	−2.2	−0.3	−7.0
**30–34**	4.1	3.7	2.6	2.7	2.0	1.0	0.22	−0.7	0.7	−7.6
**35–39**	3.0	2.9	2.6	3.5	2.6	1.7	1.39	1.0	1.6	−10.4
**40–44**	1.1	1.0	0.9	2.1	2.9	3.0	3.88	5.3	4.9	−14.2
**45–49**	3.2	3.1	2.3	2.4	2.3	2.2	2.67	3.3	3.4	−14.2
**50–54**	− 1.5	−1.8	−3.1	− 1.5	2.9	7.2	9.63	11.9	10.2	−12.8
**55–59**	−0.4	−0.1	−0.1	−0.1	1.6	4.2	5.35	6.1	5.8	−10.6
**60–64**	0.3	0.8	1.5	1.2	0.9	2.6	3.17	2.9	2.8	−7.5
**65–69**	1.1	1.3	1.5	1.1	0.1	0.4	0.70	0.5	0.2	−2.8
**70–74**	−0.2	−0.3	−0.3	−0.2	− 0.2	− 0.4	−0.47	− 0.4	−0.6	2.7
**75–79**	−0.7	−0.6	− 0.6	−1.4	−1.8	−1.4	−0.55	0.1	−2.3	6.9
**80–84**	−1.1	−0.5	−0.2	−2.4	−3.6	−1.4	0.97	2.6	−3.1	7.3
**85+**	−1.6	0.1	1.1	−4.0	−6.4	−1.1	4.4	8.0	−3.8	6.8

Source: Own calculations; base year: 2019

Overall, a large negative contribution to a rise in G_0_ and a drop in e_0_ is manifested from the age group of 35–79 years in 2020. A modest contribution of oldest of old (80+) ages attributable to COVID-19 disease is because of higher mortality rates of degenerative diseases [84, 85]. The differences in the per cent contributions between 2020 and 2019, i.e. two subsequent years, is attributable to the mortality pattern of COVID-19 disease. The decomposition analysis reveals that the age-specific contributions to ΔG_0_ and Δe_0_ in the pandemic year 2020 are discrete from those in previous years. A lesser contribution of early (0–19 years) and a larger contribution of adult (35–79 years) in the pandemic year 2020 compared to that in non-pandemic years in the studied period attest a greater role of the burden of COVID-19 disease. By gender, the outcomes reveal a larger vulnerability in men than in women.

### Age-specific contributions to sex differences in e_0_ and G_0_

The discrete decomposition method [77] and the replacement method [75] was applied for decomposing sex differences in e_0_. Results from both the discrete and replacement methods are very similar (Table 8). The interpretations from both methods remain the same.

**Table 8:** Age-specific per cent contributions to sex difference in e_0_, India, 2019–2020, replacement and discrete methods

Age Group	Non-pandemic year 2019		Pandemic Year 2020	
	Replacement method	Discrete method	Replacement method	Discrete method
**0–1**	−5.5	−5.5	−6.6	− 6.5
**1–4**	−2.7	−2.6	−1.1	−1.1
**5–9**	−0.7	−0.7	−0.7	− 0.7
**10–14**	− 0.1	− 0.1	− 0.1	−0.1
**15–19**	− 1.1	− 1.0	− 2.5	−2.4
**20–24**	1.3	1.3	1.3	1.2
**25–29**	3.4	3.3	3.2	3.1
**30–34**	6.6	6.5	7.8	7.6
**35–39**	9.3	9.1	9.5	9.4
**40–44**	8.7	8.5	8.6	8.4
**45–49**	12.4	12.2	12.6	12.3
**50–54**	7.8	7.6	7.6	7.4
**55–59**	15.9	15.4	16.3	15.8
**60–64**	11.8	11.5	12.3	11.9
**65–69**	11.6	11.1	11.3	10.8
**70–74**	8.9	9.0	7.7	8.8
**75–79**	7.5	6.8	7.2	6.7
**80–84**	2.8	4.1	3.3	4.1
**85+**	2.1	3.5	2.2	3.4

Source: Own calculations; men is the base gender

Table 9 shows the age-specific contributions to the sex differences in e_0_ during 2010–2020. The positive and negative per cent contributions of age groups to the sex differences in e_0_ are advantages and disadvantages, respectively, to women, and vice versa, as men are the base gender. The age-specific per cent contributions reveal disadvantages to females in infant through adolescent age groups and advantages to women in adult through old age groups in the entire period of 2010–2020. Children age group’s contribution showed a decline over time; otherwise, the overall contribution of 0–19 years to the sex differences in e_0_ remained more or less at − 11% in the entire period of 2010–2020. A slight increase of 1 % in infants and adolescents in 2020 versus 2019 is attributable to higher mortality rates of the COVID-19 disease in women than in men. This increase of the 1 % negative contribution of the age group of 0–19 years to sex difference in e_0_ is attributable to the higher mortality rates of COVID-19 disease in women than in men.

**Table 9:** Age-specific per cent contributions to sex differences in e_0,_ India

Age group/Year	2010	2011	2012	2013	2014	2015	2016	2017	2018	2019	2020
**0–1**	−3.2	− 3.5	− 3.8	−4.4	−5.2	−6.3	−6.1	−6.0	−5.8	−5.5	−6.6
**1–4**	−8.1	−7.7	−6.9	−6.5	−6.0	−5.4	− 4.1	− 3.4	−2.9	− 2.7	−1.1
**5–9**	0.1	0.1	0.1	−0.2	−0.5	− 0.4	−1.0	− 1.3	−0.8	− 0.7	− 0.7
**10–14**	0.9	1.0	1.0	0.5	0.7	0.5	−0.3	− 0.5	− 0.1	−0.1	− 0.1
**15–19**	−1.2	− 1.1	− 0.7	−0.6	0.2	−0.2	− 1.2	− 1.5	−0.9	−1.1	−2.5
**20–24**	0.5	0.7	1.4	1.8	1.8	1.7	1.6	1.5	1.4	1.3	1.3
**25–29**	3.6	3.5	3.4	3.3	3.3	3.2	2.7	2.6	3.2	3.4	3.2
**30–34**	4.6	4.9	5.6	5.5	5.7	6.3	6.6	6.8	6.5	6.6	7.8
**35–39**	9.2	9.1	8.6	8.1	8.6	9.3	9.2	9.2	9.2	9.3	9.5
**40–44**	9.6	9.8	10.1	9.6	8.5	7.5	8.4	9.0	8.6	8.7	8.6
**45–49**	10.2	10.6	11.8	12.6	11.9	12.9	13.3	13.2	12.4	12.4	12.6
**50–54**	10.7	11.1	12.1	11.4	8.9	6.9	5.7	5.7	7.4	7.8	7.6
**55–59**	15.1	15.4	15.6	15.0	13.9	16.5	18.0	17.9	15.9	15.9	16.3
**60–64**	13.7	13.1	11.1	9.4	10.2	11.9	12.2	12.0	11.7	11.8	12.3
**65–69**	13.7	12.7	9.9	8.8	10.3	11.5	12.0	12.1	11.6	11.6	11.3
**70–74**	8.8	8.9	9.4	9.7	9.5	8.8	9.1	9.3	9.0	8.9	7.7
**75–79**	7.1	6.9	7.1	8.6	8.6	7.6	8.4	8.8	7.8	7.5	7.2
**80–84**	2.9	2.7	2.9	5.2	6.2	4.4	3.3	3.0	3.4	2.8	3.3
**85+**	1.9	1.7	1.3	2.2	3.4	3.2	2.1	1.6	2.3	2.1	2.2

Source: Own calculations; men is reference gender; positive and negative contributions show widening and narrowing of the gender gap, respectively

The positive per cent contribution to the sex differences in e_0_ slopes up from the young-adult (20–24 years) age group. It asserts that adult- and old-age mortality rates in men were higher, including a wide range of age groups. The contribution of adult (20–64 years) age group was 79.2% to the sex difference in e_0_ in 2020. The contribution of the adult age group is considerably larger in 2020 than in 2019 and previous years. The old age group of 65+ years also contributed 31.8% to the sex differences in e_0_, which is smaller in 2020 than in 2019 and other previous years. The results of sex difference in e_0_ confirm a disadvantage in men in 20+ years regarding mortality of COVID-19 disease causing more deaths in men.

Table 10 shows the age-specific per cent contributions to sex differences in G_0_ in 2010–2020. Infant and adolescent age groups contributed − 33 and − 11% in 2020, which are larger than in recent years. Children’s age group only showed a decline in contribution over time to − 8.4% in 2020. The negative per cent contributions of infant, child, and adolescent age groups confirm an equalising effect on G_0_ by the higher burden of COVID-19 mortality, more in females than in males. The age group of 0–19 years contributed − 52.3% to the sex differences in G_0_, which is considerably larger than that in recent past years but lies in trends with the early 2010s.

**Table 10:** Age-specific per cent contributions to sex differences in G_0,_ India

Age group/Year	2010	2011	2012	2013	2014	2015	2016	2017	2018	2019	2020
**0–1**	−19.5	−19.0	−15.2	−19.3	−28.6	−34.3	−29.3	−26.0	−24.3	−21.7	−33.0
**1–4**	−40.8	−35.6	−27.2	−29.0	−29.7	−25.7	− 18.1	− 14.0	− 11.3	−9.7	−5.1
**5–9**	0.3	0.3	0.2	−0.8	−2.6	− 2.0	−4.2	−5.1	− 3.0	− 2.6	− 3.2
**10–14**	4.1	4.4	3.8	2.0	3.1	2.1	−1.1	− 1.8	− 0.2	− 0.5	− 0.6
**15–19**	− 5.4	−4.5	− 2.4	−2.3	1.0	− 0.8	−4.8	− 5.6	−3.1	− 3.5	− 10.4
**20–24**	2.3	2.8	5.0	7.1	8.1	7.3	6.3	5.6	5.0	4.3	5.1
**25–29**	15.1	13.6	11.3	12.6	14.1	12.9	10.1	9.1	10.7	10.6	12.5
**30–34**	18.4	18.1	17.8	20.0	23.2	24.4	23.8	22.8	20.5	19.4	28.5
**35–39**	34.1	31.5	25.3	27.2	32.6	33.7	30.8	28.8	27.1	25.5	32.4
**40–44**	32.5	30.9	27.1	29.5	29.6	24.8	25.6	26.1	23.3	21.9	26.5
**45–49**	30.2	29.2	28.1	34.4	36.6	37.8	36.2	33.9	29.9	28.0	33.8
**50–54**	26.5	25.5	23.9	26.2	23.2	16.9	13.0	12.2	15.0	14.9	16.7
**55–59**	27.8	26.2	22.9	26.3	28.3	31.1	31.4	29.8	25.2	23.8	26.5
**60–64**	14.3	12.6	9.4	10.2	13.4	14.1	13.3	12.7	12.1	11.6	11.3
**65–69**	1.2	0.7	0.8	2.2	4.5	4.0	3.7	3.8	4.0	3.9	0.6
**70–74**	−8.7	−8.4	−7.4	−7.2	−6.2	−5.8	−5.8	−5.3	−4.3	−3.8	− 7.3
**75–79**	−15.2	− 13.9	− 12.0	− 15.6	−16.3	−14.0	−14.1	−13.7	−11.1	−9.8	− 14.8
**80–84**	−9.3	−8.0	−7.3	− 15.3	− 20.1	− 13.8	−9.4	−7.9	− 8.2	− 6.3	− 10.5
**85+**	− 8.0	−6.3	−4.3	− 8.1	− 14.0	− 12.8	− 7.4	− 5.3	− 7.3	− 6.1	− 8.8

Source: Own calculations; men is reference gender; positive and negative contributions show widening and narrowing of the gender gap, respectively

The adult age group showed positive contributions to the sex differences in G_0_. The middle-aged adult age group contributed 92.6%, followed by older-adult and young-adult age groups of 54.5 and 46.0% to the sex difference in G_0_. The old age groups of 65–79 years and 80+ years contributed − 21.5 and − 19.3% to the sex difference in G_0_. It confirms a higher mortality rate in men than in women. The disequalising effect was stronger in adult ages in 2020 when compared to 2019 and previous years, more likely in men than in women. The contributions of adult (20–64 years) and old (65+ years) age groups in 2020 are distinguishable from those in 2019 and previous years. While the early and adult age groups have a disequalising effect on ΔG_0_, the old age group has an equalising effect on ΔG_0_. Given that, the negative per cent contributions in the old age group show a more equalising effect on ΔG_0_ by women than by men. Altogether, men compared to women show a disadvantage in mortality because of a less equalising effect in old ages as well as a large disequalising effect on ΔG_0_. It led to higher G_0_ values in 2020 versus in 2019. Overall, the outcomes of sex difference in G_0_ furthermore confirms a vulnerability of men regarding COVID-19 mortality.

The analyses of age-specific contributions to the sex differences in e_0_ and G_0_ point out that the adult (20–64 years) age group in which mortality rates of COVID-19 disease significantly contributed to the sex differences in e_0_ and G_0_. The trends in age-specific contributions reveal a wider sex difference in G_0_ in the pandemic year 2020, which is distinguishable but not disparate from the past years during 2010–2020. Whereas the age-specific contributions to Δe_0_ between the pandemic year 2020 and the base year 2019 are discrete from previous years during 2010–2020, showing the burden of COVID-19 disease in many age groups. The comparison of age-specific contributions to Δe_0_ and ΔG_0_ and sex differences in e_0_ and G_0_ during 2010–2020 highlights the burden of COVID-19 mortality in men aged 35–64 years and 65–79 years as compared to women. The outcomes confirm that the burden of COVID-19 disease has amplified mortality rates more disproportionally in men than in women.

### Contributions of causes of death including COVID-19 disease in Δe_0_ and ΔG_0_, 2010–2020

Tables 11 and 12 show the per cent contributions of COVID-19 disease and other causes of death to sex differences in e_0_ and G_0_, respectively. The positive and negative age-specific per cent contributions of causes of death to sex difference in e_0_ reveal the share of major causes of death responsible for the gender gap in e_0_. In comparison, the positive and negative age-specific per cent contributions of causes of death to sex difference in G_0_ reveals the share of causes of death responsible for widening and narrowing, respectively, the gender gap.

**Table 11:** Per cent contributions to sex difference in e_0_ by 22 causes of death, India, 2010–2020

Causes of death/Year	2010	2011	2012	2013	2014	2015	2016	2017	2018	2019	2020
**Cardiovascular diseases**	41.7	42.6	41.7	42.0	42.0	43.6	44.7	44.6	43.8	44.0	44.6
**Chronic respiratory diseases**	13.9	13.6	13.1	14.3	15.4	15.5	14.9	14.7	14.3	13.8	13.5
**COVID 19 disease**	**0.0**	**0.0**	**0.0**	**0.0**	**0.0**	**0.0**	**0.0**	**0.0**	**0.0**	**0.0**	**−1.5**
**Diabetes and kidney diseases**	5.9	5.9	5.7	5.7	5.6	5.7	6.0	6.1	6.1	6.2	6.1
**Digestive diseases**	13.4	13.6	13.4	13.6	14.0	15.6	15.7	15.7	15.3	15.4	16.0
**Enteric (Diarrhea and Typhoid) infections**	−11.8	−12.0	−11.5	−12.6	−14.5	− 16.3	−16.1	− 16.2	−14.7	− 14.3	−14.1
**HIV/AIDS and sexually transmitted infections**	0.6	0.3	0.2	0.1	0.1	0.1	0.1	0.1	0.1	0.0	0.0
**Maternal and neonatal disorders**	−3.1	−3.0	−2.6	−2.7	−2.7	−3.1	−3.4	−3.5	−3.8	−3.9	−4.2
**Mental disorders**	0.0	0.0	0.0	0.0	0.0	0.0	0.0	0.0	0.0	0.0	0.0
**Musculoskeletal disorders**	−0.3	−0.3	−0.3	−0.4	−0.4	−0.5	−0.6	−0.6	− 0.6	− 0.7	− 0.6
**Neglected tropical diseases and malaria**	0.4	0.2	0.3	0.3	0.3	0.3	0.2	0.2	0.3	0.3	0.3
**Neoplasms**	4.7	4.6	4.4	3.5	2.3	1.4	1.5	1.5	1.6	1.7	1.2
**Neurological disorders**	0.7	0.7	0.7	0.8	0.8	0.8	0.6	0.6	0.6	0.6	0.6
**Nutritional deficiencies**	−2.0	−2.0	−1.9	−1.9	−1.9	−2.0	−1.8	−1.6	−1.4	−1.4	−1.5
**Other infectious diseases**	−2.6	−2.6	− 2.3	−2.1	− 2.0	−1.8	− 1.6	−1.2	−0.9	−0.8	−0.9
**Other non-communicable diseases**	−0.5	− 0.6	− 0.6	−0.8	− 1.0	− 1.3	−1.3	− 1.4	−1.3	− 1.3	−1.5
**Respiratory infections and tuberculosis**	11.0	10.3	11.2	11.7	12.6	11.4	11.6	12.0	11.2	10.6	10.4
**Self-harm and interpersonal violence**	4.5	4.6	4.7	4.8	5.1	5.3	5.3	5.2	5.3	5.2	5.4
**Skin and subcutaneous diseases**	0.0	0.0	0.0	0.0	0.0	−0.1	0.0	0.0	0.0	0.0	−0.1
**Substance use disorders**	2.2	2.3	2.2	2.4	2.5	2.9	2.9	3.0	2.9	3.0	3.2
**Transport injuries**	15.9	16.3	16.3	16.4	16.9	17.8	17.3	17.0	17.3	17.5	18.6
**Unintentional injuries**	5.5	5.4	5.4	5.3	4.8	4.8	4.0	3.7	4.0	3.9	4.3

Source: Own calculations; men is the reference gender

**Table 12:** Per cent contributions to sex difference in G_0_ by 22 causes of death, India, 2010–2020

Causes of death/Year	2010	2011	2012	2013	2014	2015	2016	2017	2018	2019	2020
**Cardiovascular diseases**	46.2	45.3	40.8	43.6	46.6	49.3	47.8	46.2	42.7	41.6	45.9
**Chronic respiratory diseases**	−2.7	−1.8	−0.8	−4.4	−7.6	−5.7	−3.0	−2.5	−2.0	−0.6	−4.1
**COVID 19 disease**	**0.0**	**0.0**	**0.0**	**0.0**	**0.0**	**0.0**	**0.0**	**0.0**	**0.0**	**0.0**	**−9.5**
**Diabetes and kidney diseases**	3.4	3.5	3.4	2.7	1.5	1.2	1.5	1.7	1.7	1.9	1.5
**Digestive diseases**	29.2	27.6	23.7	27.6	32.2	33.8	31.4	29.9	27.9	26.4	32.5
**Enteric (Diarrhea and Typhoid) infections**	−20.6	−17.3	−12.7	−16.1	−18.6	−16.1	−12.3	− 11.0	−8.1	−6.4	−8.5
**HIV/AIDS and sexually transmitted infections**	1.9	0.8	0.3	0.2	0.5	0.3	0.1	0.3	0.2	0.0	0.0
**Maternal and neonatal disorders**	−8.6	−7.8	−5.5	−6.8	−7.7	−9.0	−10.0	−9.9	−10.7	− 10.4	−13.6
**Mental disorders**	0.0	0.0	0.0	0.0	0.0	0.0	0.0	0.0	0.0	0.0	0.0
**Musculoskeletal disorders**	−0.1	−0.1	0.0	−0.2	−0.3	−0.2	−0.2	−0.2	−0.2	−0.2	−0.1
**Neglected tropical diseases and malaria**	−2.2	−2.3	0.6	1.5	−1.2	−2.9	−1.2	−0.2	−0.9	− 0.9	− 1.2
**Neoplasms**	−7.4	−7.1	−5.1	−8.9	−14.4	− 17.7	−16.8	− 15.9	−14.0	− 12.8	−17.6
**Neurological disorders**	0.4	0.3	0.3	−0.2	− 0.5	− 0.5	− 0.6	−0.5	− 0.3	−0.2	− 0.4
**Nutritional deficiencies**	− 8.7	− 7.8	−6.4	−7.3	− 8.2	− 7.8	− 6.1	− 5.0	−4.2	−3.7	− 5.1
**Other infectious diseases**	−14.3	− 12.9	−9.6	− 10.4	− 10.6	− 9.3	− 7.7	− 5.7	− 4.1	− 3.3	− 4.7
**Other non-communicable diseases**	− 4.9	− 4.8	−4.1	− 5.6	− 7.4	− 8.3	− 7.8	− 7.4	− 6.5	− 6.0	− 8.4
**Respiratory infections and tuberculosis**	− 2.4	− 2.4	0.9	− 0.2	− 0.9	− 4.2	− 0.7	1.2	1.6	1.7	−1.6
**Self-harm and interpersonal violence**	11.1	10.7	9.7	11.5	14.3	13.9	12.5	11.4	11.1	10.0	12.6
**Skin and subcutaneous diseases**	−0.7	− 0.7	− 0.5	−0.7	− 0.8	−0.9	− 0.8	−0.7	− 0.6	−0.6	− 0.8
**Substance use disorders**	5.9	5.6	4.7	5.7	6.8	7.3	6.9	6.6	6.2	5.9	7.5
**Transport injuries**	53.2	50.8	43.4	49.6	56.9	56.7	50.1	46.4	44.9	42.5	56.1
**Unintentional injuries**	21.5	20.3	17.0	18.3	19.5	20.3	16.8	15.5	15.4	14.8	19.4

Source: Own calculations; men is the reference gender

The largest contribution of cardiovascular disease to the sex difference in e_0_ and G_0_ was 44.6 and 45.9%, respectively, in 2020. It confirms the higher toll of deaths in men compared to women. Cardiovascular disease is strongly responsible for the sex difference in e_0_ during the period 2010–2020. Notably, the cardiovascular disease shows the strongest role in widening the sex difference in G_0_ during 2010–2020. Noncommunicable diseases such as chronic respiratory diseases contribute to 13.5 and − 4.1% of the sex difference in e_0_ and G_0_, respectively. While chronic respiratory disease also confirms a significant role for the gender gap in e_0_ but a minor role for narrowing the sex difference in G_0_ compared to other noncommunicable diseases. Digestive diseases, enteric infections, respiratory infections and tuberculosis, and transport injuries contributed considerably to sex differences in e_0_ and G_0_. Altogether, noncommunicable diseases contributed 93.5 and 54.5% to the sex differences in e_0_ and G_0_, respectively, and are majorly responsible for widening the sex differences in e_0_ and G_0_.

On the other hand, communicable diseases contributed − 21.9 and − 42.6% to the sex differences in e_0_ and G_0_, respectively. Communicable diseases are majorly responsible for narrowing the sex difference in e_0_ and G_0_. Among communicable diseases, enteric infections, and respiratory infections and tuberculosis show a large and significant share of contributions to the sex differences in e_0_ and G_0_. Amongst communicable diseases, the COVID-19 disease shows a significant contribution of − 1.5 and − 9.5% to the sex differences in e_0_ and G_0_, respectively.

The COVID-19 disease shows a negative repercussion to the sex differences in e_0_ and G_0_. This disadvantage for men compared to women is evident in lower e_0_ and higher G_0_ in 2020. By lowering e_0_ and increasing G_0,_ the COVID-19 disease is accountable for destabilising the coherent progress of e_0_ and G_0_, favouring women. With the increase in inequality in age at death, both women and men lost the gain in e_0_ and G_0_ achieved during the recent past years.

## Discussion

The study explores the repercussions of the mortality pattern of COVID-19 disease as one of the causes of death [38] on the life expectancy at birth (e_0_) and inequality in age at death (G_0_) [74, 76] for India in the entire period of 2010–2020. The study examines the changes in e_0_ and G_0_ for both sexes by assessing the age-specific contributions of mortality patterns, including that of COVID-19 disease [75, 77], using data from GBD [36] and COVID19-India API [12]. The study reckons the contribution of the COVID-19 disease and many causes of death to the sex differences in e_0_ and G_0_, focusing on the pandemic year 2020 versus the non-pandemic year 2019.

The age pattern of mortality of COVID-19 disease reveals that the gradient of mortality slopes in adult ages [43, 80] and increases exponentially in old ages, more accelerating in men than in women (Figs. 1, 2 and 3). The life table estimates [33, 34, 74] in 2020 versus 2019 (Table 2) reveals a drop of 2.0 and 2.3 years in e_0_ for men and women, respectively. The age group of 35–79 years (Tables 4 and 5) mainly explains the Δe_0_. The drop in e_0_ values is attributable to the excess deaths caused by COVID-19 disease [4, 86]. This reduction in e_0_ manifests the retrograding progress in India’s mortality transition [18, 25, 32, 43, 87]. The COVID-19 disease in India shows a potential to cancel the gain in e_0_ by six to eight years (Fig. 4) [16, 39, 40, 88, 89].

The repercussions of the excess mortality of COVID-19 disease is evident [90] for inequality in age at death [16]. The G_0_ values of men and women increased from 0.150 and 0.141 in 2019 to 0.159 and 0.153, respectively, in 2020 (Fig. 4). The age group of 20–64 years contributed remarkably to the dispersion in age at death (Tables 6 and 7). A large dispersion in age at death demonstrates a high heterogeneity in the mortality pattern of COVID-19 disease, more strongly in men than in women. Therefore, the burden of COVID-19 disease has a severe impact on the inequality in age at death. The trends in G_0_ for India shows a consistent decline during 2010–2019. However, the burden of COVID-19 disease has not only affected the dispersion in age at death but also shows an uptick in G_0_ value in 2020. It has affected the inequality trends in mortality for at least five years. An uptick in the G_0_ is in agreement with a diminution of e_0_, as corroborated by the phenomenon of high e_0_ and low G_0_ [42, 91].

The excess mortality of COVID-19 disease led to the sex differences in e_0_ and G_0_ contributed by many age groups [41]. The decomposition analyses of the sex differences in e_0_ and G_0_ reveal that the age-specific contributions in 2020 are distinguishable from those in previous years. Adult and old age groups are significant contributors to the gender gap in e_0_ and G_0_ (Tables 9 and 10). In addition to the decomposition analyses of e_0_ and G_0_ over time, the analysis of sex differences in e_0_ and G_0_ also confirms a major contribution from the age group of 35–79 years. The sex differences in e_0_ and G_0_ are negatively skewed towards men. The COVID-19 disease contributed − 1.5 and − 9.5% to the sex differences in e_0_ and G_0_. The negative contribution and disequalising effect of COVID-19 disease explain more deaths in men compared to women. The disequalising effect of COVID-19 disease is in congruence with that of communicable diseases. Communicable disease contributes advantageously to narrow the gender gap in mortality. In contrast, the noncommunicable disease with its the largest share of the sex difference in e_0_ and G_0_ contributes disadvantageously to widen the gender gap in mortality [73, 92]. Altogether, analyses of sex differences in e_0_ and G_0_ confirms that the vulnerability of men in pandemic time gets amplified more in men than in women [10, 15, 93–96], attributable to the burden of COVID-19 disease, which is in addition to the higher mortality rates in men than in women in the past.

The mortality pattern of COVID-19 disease, the age-specific contributions to Δe_0_ and ΔG_0_, and the sex difference in e_0_ and G_0_ confirm a more significant role of the age group of 35–79 years which is almost two to three folds larger than in past years during 2010–2019. Adult and young-old age groups explain a large dispersion in age at death. It marks that deaths that occurred in the age group of 35–79 years in the pandemic year 2020 were significantly excess of the toll of deaths in normal or previous years. Importantly, deaths caused by COVID-19 disease in adult and young-old age groups were unevenly distributed. Overall, the ongoing pandemic of COVID-19 disease has halted the progress in the secular trend of life expectancy at birth and inequality in age at death [11] in India.

## Limitations of the study

The deceased and confirmed cases of COVID-19 disease is available at the state level and district level [12, 36]. However, ASDRs calculated at these lower levels of geography are unreliable because of a large missing age-sex mortality data for many cases. At the national level, the age-sex mortality data allows the calculation of age-specific death rates.

Explicit information on symptomatic plus asymptomatic carriers of the SARS-CoV-2 virus is not available in various sources of data related to COVID-19 disease. So, in the lack of that, an average seroprevalence based on the three seroepidemiological surveys in India [54–56] were used for the study. We have noticed the use of different assays used in the seroepidemiological surveys. The first seroepidemiological survey used ELISA IgG assay; however, we considered that in the knowledge of 0.73% seroprevalence in 18+ years population which did not much affect an average value. The third seroepidemiological survey was between 17 Dec 2020 to 08 Jan 2021; however, we have considered it for adjustment of seroprevalence. The estimates of seroprevalence at national, regional and local level surveys vary widely [58]. Also, most of the surveys have considered 0–17 years as the lowest age group. Nevertheless, most of the surveys found no significant difference in seroprevalence across the age groups. So, for the children’s (0–9 years) age group, we have assumed the same seroprevalence as that of the 10–17 years age group at the national level.

## Conclusion

The study demonstrates the impact of the excess mortality of COVID-19 disease on e_0_ and G_0_ in India. The mortality pattern of COVID-19 reveals a drop of 2.0 and 2.3 years for men and women, respectively, between the pandemic year 2020 and the non-pandemic year 2019. Analogously, the inequality in age at death of COVID-19 disease increased in 2020 as compared to 2019. A drop in e_0_ and rise in G_0_ is significantly contributed by the age group of 35–79 years. This age group of 35–79 years marks excess deaths caused by COVID-19 disease in 2020 compared to normal years and contributed remarkably to the sex differences in e_0_ and G_0_. The COVID-19 disease demonstrates its potential to cancel the gains of six to eight years in life expectancy at birth and five years in inequality in age at death. The COVID-19 pandemic has negative repercussions on life expectancy and inequality in age at death and has slowed the mortality transition in India.

## Data Availability

The datasets generated and/or analysed during the current study are available in the COVID19-India Application Programming Interface (API) portal repository, India, https://api.covid19india.org/ [12] and Global Burden of Disease Study 2019 (GBD 2019), Institute for Health Metrics and Evaluation (IHME), United States, http://ghdx.healthdata.org/gbd-results-tool [36].

## Abbreviations

SRS: Sample Registration System
COVID-19: Coronavirus disease of 2019
SARS-CoV-2: Severe Acute Respiratory Syndrome Coronavirus 2
GBD: Global Burden of Disease
ORG&CC: Office of the Registrar General & Census of Commissioner
MoHFW: Ministry of Health and Family Welfare
IHME: Institute for Health Metrics and Evaluation

## Footnotes

1 The total number of deaths in 2019 is assumed as the same as that in the pandemic year 2020.

2 The descriptions of age groups or age-specific contributions are presented by the infants (0–1 year), children (1–9 years), adolescents (10–19 years), young adults (20–34 years), middle-aged adults (35–49 years), older adults (50–64 years) and young olds (65–79 years) and oldest of olds (80+ years). The age groups of 0–19 years, 20–64 years, and 65+ years are presented as the early, adult, and old age groups, respectively.

3 Confidence intervals of e_0_ were computed by a standard method [81]
